# Perfectionism in Anorexia Nervosa: Novel Performance Based Evidence

**DOI:** 10.1371/journal.pone.0111697

**Published:** 2014-10-31

**Authors:** Samantha Lloyd, Jenny Yiend, Ulrike Schmidt, Kate Tchanturia

**Affiliations:** 1 King's College London, Department of Psychological Medicine, Institute of Psychiatry, London, United Kingdom; 2 King's College London, Department of Psychosis Studies, Institute of Psychiatry, London, United Kingdom; 3 South London and Maudsley NHS Foundation Trust, London, United Kingdom; 4 Illia State University, Tbilisi, Georgia, United States of America; University College London, United Kingdom

## Abstract

Existing research into perfectionism in Anorexia Nervosa (AN) is limited by a reliance upon self-report measures. This study used novel performance based measures to investigate whether there is behavioural evidence for elevated perfectionism in AN. 153 participants took part in the study – 81 with a diagnosis of AN and 72 healthy controls (HCs). Participants completed two performance based tasks assessing perfectionism – a text replication task and a bead sorting task – along with self-report measures of perfectionism. Significant group differences were observed on both tasks. In the text replication task the AN group took significantly longer compared with healthy controls (p = 0.03, d = 0.36) and produced significantly higher quality copies (p = <0.01, d = 0.45). In the bead sorting task, there was a trend towards more participants in the AN group choosing to check their work compared with the HC group (p = 0.07, d = 0.30) and the AN group took significantly longer checking than those in the HC group (p = <0.01, d = 0.45). Only copy quality uniquely predicted scores on self report measures of perfectionism. This study provides empirically tested evidence of elevated performance based perfectionism in AN compared with a healthy control group.

## Introduction

Anorexia nervosa (AN) is an eating disorder characterized by restriction of energy intake leading to a significantly low body weight; intense fear of gaining weight, or persistent behaviour that interferes with weight gain; and disturbance in the way in which one's body weight or shape is experienced, undue influence of body weight or shape on self-evaluation, or persistent lack of recognition of the seriousness of the current low body weight [1]. Perfectionism has been implicated as both a risk and maintaining factor for AN [2]–[5]. It is a multi-dimensional personality feature or temperament characterised by the setting of extremely high and demanding performance standards, which an individual with perfectionism strives for and bases their self-evaluation on. Perfectionism is conceptualised as a transdiagnostic maintaining mechanism due to its implication as a risk factor in a range of disorders [6], [7]. Consistent evidence has been found for elevated self-reported perfectionism in individuals with eating disorders [6], [8], [9] and there is evidence that levels of perfectionism are elevated in eating disorders relative to other disorders [8]. This has led some researchers to propose that eating disorder symptoms are an expression of perfectionism specifically in the areas of eating, shape and weight, rather than simply occurring comorbidly [10], [11]. There is also some evidence that perfectionism impacts upon treatment outcome, with a number of studies having found this trait to be predictive of poorer prognosis and treatment drop-out [12]–[14]. Based upon the above, perfectionism has been identified as a potential target for interventions in AN [6].

Despite considerable research into perfectionism in AN, existing research is limited by a reliance upon self-report measures and there is a lack of empirical or behavioural evidence for elevated perfectionism in AN. Only one study [15] has experimentally measured perfectionism in relation to disordered eating, with this study carried out with a non-clinical sample. In comparing weight concerned and non-weight concerned participants, Pliner and Haddock (1996) found that during a creativity task those who scored higher on eating disorder symptoms were more likely to persist in accepting unrealistically high standards imposed by the experimenter. To our knowledge, no other studies to date have investigated perfectionism in AN relative to healthy controls using objective experimental measures of perfectionist behaviours.

Whilst self-report measures and clinical experience support the presence of elevated perfectionism in AN, empirical studies investigating the behavioural evidence for elevated perfectionism are required for a number of reasons. Accurate reporting on self-report measures requires a level of insight and acceptance of illness. Our clinical experience suggests that AN patients may underestimate the degree of their perfectionism, with this trait often being ego-syntonic in nature. Research evidence also suggests that denial of illness is a pertinent issue in eating disorders [16]. Furthermore, there is evidence that self-report measures do not necessarily correlate with performance based measures. In AN studies, Lounes, Khan & Tchanturia [17] found low correspondence between self-reported cognitive flexibility and performance on an experimental measure of flexibility, whilst discrepancies have also been found between self-report and empirical measures of certain eating disorder symptoms [18]. Discrepancies between self report and empirical measures are also common in research generally. For example, discrepancies have been identified between self estimated and task performance based measured cognitive abilities [19]. These discrepancies may extend to other self-report constructs and their behavioural equivalents, including perfectionism. Empirical behavioural measures have the potential to clarify the current ambiguity relating to the use of self report measures in eating disorders and the degree to which perfectionism is helpful or harmful in eating disorders.

This study aimed to use novel experimental paradigms in order to investigate whether there is behavioural evidence for elevated perfectionism in individuals with AN, as well as investigating associations between performance based and self-report measures of perfectionism. The study achieved these aims.

## Materials and Methods

### Participants

**Figure 1:**
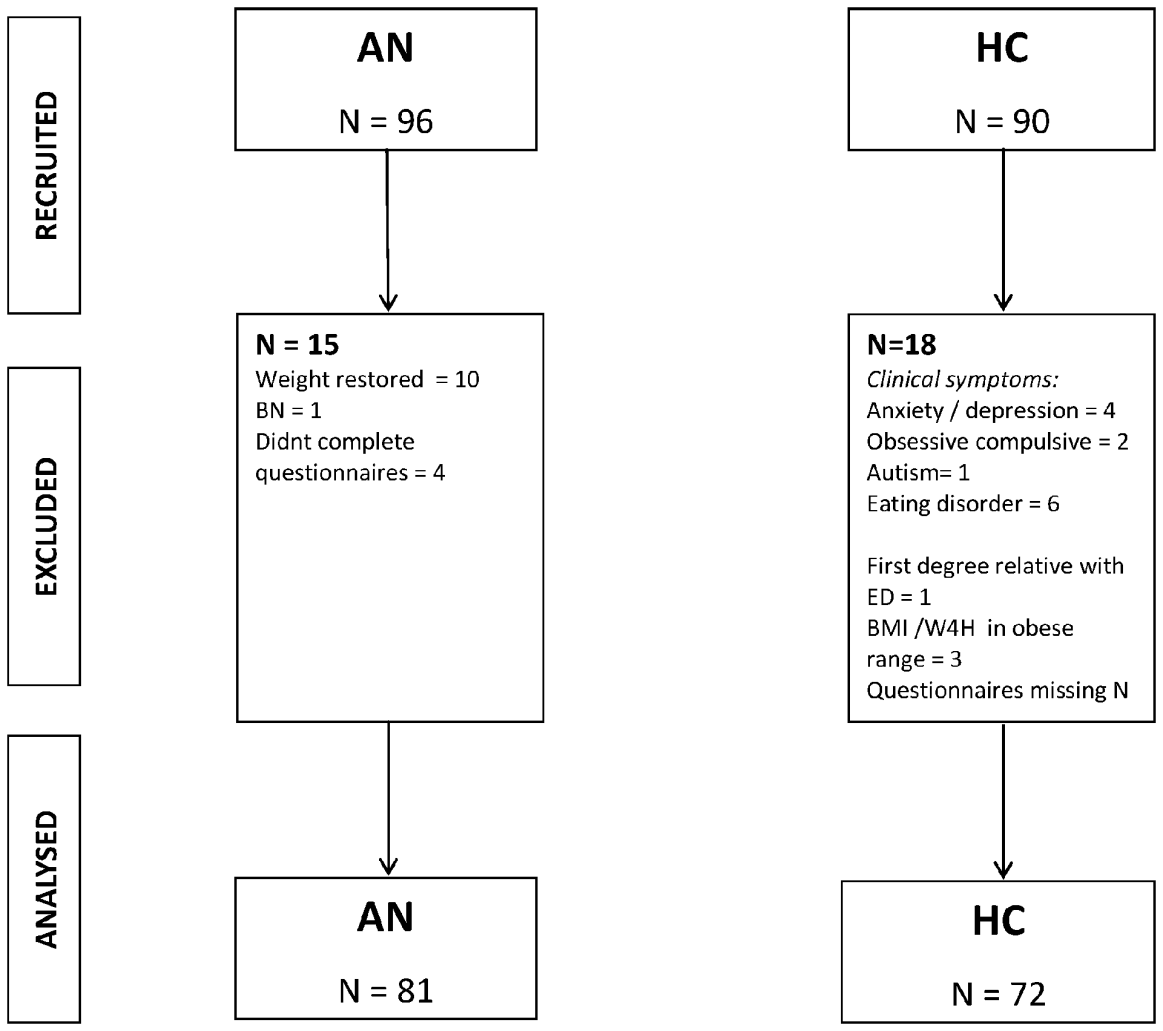
Flow of participants through study.

153 participants took part in the study – 81 with a primary clinical diagnosis of AN and 72 healthy controls (HCs). Inclusion criteria for AN participants were a formal diagnosis of AN according to either DSM-IV or DSM-V criteria and a BMI of below 17.5 for adults or percentage weight for height of below 90% for adolescents. AN diagnosis was confirmed using the SCID Structured Clinical Interview (research version) for DSM-IV (SCID; [20]). Inclusion criteria for HCs were a BMI of above 18.5 in adults or percentage weight for height of above 90% if aged 18 or lower, no past or current eating disorder symptoms or other psychiatric disorder and no family history (first degree relatives) of eating disorders. Presence of psychiatric symptoms was assessed using scores on psychometric measures (see measures section) and the SCID. Inclusion criteria for both groups were being aged between 11 and 55 and fluency in English. HCs were excluded if obese – a BMI of 30 or above in adults and a percentage weight for height of above 120% in participants 18 or under. Exclusion criteria for both groups were a diagnosis of a psychotic disorder or a severe medical disorder. HCs were excluded if there was a family history of eating disorders due to evidence of first degree relatives of those with eating disorders having elevated levels of traits such as perfectionism and inflexibility [9], [21]. AN participants were recruited from adult and adolescent eating disorder specialist services, mental health charities and self-help groups. HCs were recruited through university circulars, local online forums, adverts and schools. Figure 1 shows the flow of participants through the study including numbers recruited, excluded and analysed, along with reasons for exclusions.

### Ethics Statement

Full ethical approval for this study was granted by the Dulwich NRES Committee, London (12/LO/2013). Written informed consent was obtained from all participants.

## Measures

### Perfectionism: self-report

*Frost Multi-Dimensional Perfectionism Scale* [22]. This is a 35-item self-report measure which assesses the following aspects of perfectionism: concern over mistakes, personal standards, doubts about actions, parental criticism, parental expectation and organisation. For the purpose of this study the Concern over Mistakes, Doubts about Actions and Personal Standards subscales were used. This is a widely used measure with good psychometric properties [22], [23].

The *Clinical Perfectionism Scale; CPQ*, [24] assesses cognitive, behavioural and affective aspects of perfectionist goal setting, striving and consequences for self-evaluation. There is currently no established clinical cut-off for this measure. The measure has been found to have adequate reliability and validity [25].

### Behavioural/experimental tasks

*The text replication task*, designed by Yiend and colleagues [26] measures attention to detail, spatial organization and thoroughness when reproducing complex information. Participants were asked to copy a passage of text and a complex geometric figure as neatly, clearly and accurately as possible. Participants were given as long as they needed to complete the task and were timed, with a range of tools provided to carry out the task (paper, ruler, eraser, coloured pencils, protractor and compass). Dependent measures were time taken to complete the task and independent rating of copy quality. For scoring purposes the copy stimulus was divided into five elements, with three rating criteria relating to each. For each criterion a score between one (strongly disagree) and seven (strongly agree) was awarded based upon the extent to which participants accurately replicated an element, for example, ‘The title matches the font (size, letter form)’. A mean item rating was then calculated. Ratings were made by one rater, with a purposive sample of copies scored by a second rater blind to group.

*The bead sorting task* has previously been used to measure checking behaviour in clinical disorders associated with perfectionism [27]. Participants were given 40 coloured beads (5 beads of 8 different colours) which they were asked to sort into 12 empty narrow-necked bottles. Participants were instructed to pick up one bead at a time and put each different type of bead into a different bottle as quickly but accurately as possible within a minute. The bottles were semi-transparent, therefore making it difficult to see whether errors had been made during sorting. Once they had completed the task, participants were given the option to check for and correct any mistakes. Dependent measures were the number of beads remaining after 60 seconds, the number of participants in each group choosing to check and time spent checking. Both experimental tasks have been found to distinguish between high and low perfectionists in a non-clinical sample [26]. In order to control for any group differences in general performance a control task was used in which participants were presented with 40 beads of the same colour which they were asked to insert into one container as quickly as possible.

### Clinical and demographic measures

*The Eating Disorder Examination Questionnaire (EDE-Q*; [18]) is a self-report measure which examines cognitions and behavioural features of eating disorders and is made up of a number of subscales: dietary restraint, eating concern, shape concern and weight concern. A mean item score was derived by combining the 22 items forming these four subscales. Clinical criteria cut-off adopted was a score of higher than 2.7 on the global scale [28], [29]. Based upon the Eating Disorder Examination (EDE; [30]), the EDE-Q represents a less resource and intensive method for assessing eating disorder symptomatology. The EDE-Q has been found to have acceptable to good validity and reliability [29], although some key differences between the performance of the EDE and the EDEQ have been found. Whilst the two measures have been found to perform similarly with respect to behavioural features of disordered eating, higher scores on the EDEQ have been found for more complex features such as shape concern [18], [29].

*Hospital Anxiety and Depression Scale (HADS*; [31]). This is a widely used measure of anxiety and depression. Ranges are given for non-clinical (0–7), borderline (8–10) and probable (11–21) anxiety and depression scores. HC participants were excluded from the analysis if they had a score of above ten on either anxiety or depression (N = 4). The measure has been found to have good validity and reliability [31], [32].

*The Obsessive Compulsive Inventory – Revised (OCI-R*; [33]) is a measure of obsessive compulsive disorder (OCD) symptoms. A score of 21 is indicative of possible clinical symptom severity [33]. Studies have shown the OCI-R to be a valid and reliable measure [33], [34]. The OCI-R was used to assess OCD symptoms in adult participants.

*Children's Obsessional Compulsive Inventory* (ChOCI; [35]). This is a measure of obsessive compulsive symptoms in children and adolescents. A total score of above 17 is considered clinically significant [35]. The measure has good psychometric properties [35], [36]. The ChOCI was used to assess OCD symptoms in adolescent participants.

*Structured Clinical Interview for DSM-IV Axis I disorders (SCID-I*; [37]). This was used in conjunction with self-report measures to investigate the presence of psychiatric symptoms. The *Wechsler Abbreviated Scale of Intelligence (WASI-II*; [32]) was used to measure full scale IQ. This is made up of four subtests – block design, matrix reasoning, similarities and vocabulary – which form two subscales and from which verbal, performance and full scale IQ scores are calculated.

## Analysis

Analysis was carried out using SPSS version 21 [38] and Stata version 12 [39]. Data were checked for normality using Kolmogrov-Smirnov tests and visual inspection of histograms for continuous variables. Number of beads remaining and time taken to check on the bead sorting task were found to be non normally distributed. Descriptive analyses and regression were carried out to compare AN and HC groups on demographic and clinical variables. Group differences on normally distributed continuous data were investigated using linear or logistic regression. Quantile regression was used to analyse non normally distributed variables. Group differences on dichotomous variables were analysed using chi square. Associations between self-report and behavioural measures were investigated using Pearson's or Spearman's rho correlations as appropriate. In order to investigate independent associations between performance based and self-report measures of perfectionism, regression analyses were carried out for the AN group with each self report scale (FMPS CM, DA, PS; CPQ) entered separately as an outcome variable and all performance based measures as predictor variables. For all analyses, Cohen's d was calculated as a measure of effect size for each analysis (the difference between pre- and post-intervention scores divided by the pooled standard deviation). The following values correspond to relative effect sizes: small (d = ≥0.20 and <0.50), medium (d = ≥0.50 and <0.80) and large (d = ≥80).

## Results

### Demographic and clinical variables

AN and HC groups were matched in age. As expected the AN group were significantly lower on Body Mass Index for those aged 19 or above and percentage weight for height for younger participants. The AN group has significantly higher scores on anxiety, depression, eating disorder symptoms and obsessive compulsive symptoms. The AN group had a median illness duration of 3.0 years (IQR = 1, 6; range = 35.5). Table 1 shows demographic and clinical characteristics for each group, along with results of group comparisons.

**Table 1:** Demographic and clinical characteristics.

	AN (N = 81)	HC (N = 72)	t	B	p	ES
**Age**	21.14 (7.94)	19.54 (6.59)	1.34	1.60	0.18	-
**BMI (aged >18)**	15.20 (1.63)	21.19 (2.23)	−13.3	−6.00	<0.01	3.01
**Percentage weight for height (aged ≤18)**	80.51 (6.55)	100.30 (7.84)	−12.12	−19.80	<0.01	2.81
**IQ (WASI score)**	107.82 (12.59)	105.17 (10.08)	1.42	2.65	0.16	-
**EDEQ global score**	3.83 (1.54)	0.98 (0.73)	14.33	2.86	<0.01	2.32
**FMPS CM**	32.74 (9.17)	21.02 (6.05)	9.39	11.72	<0.01	1.52
**FMPS DA**	14.77 (3.41)	10.30 (3.34)	8.11	4.48	<0.01	1.31
**FMPS PS**	28.45 (6.08)	22.79 (5.44)	6.06	5.66	<0.01	0.98
**FMPS CPQ**	32.38 (7.55)	23.94 (5.35)	7.84	8.44	<0.01	1.27
**HADS anxiety**	13.23 (4.98)	4.68 (2.64)	12.94	8.55	<0.01	2.10
**HADS depression**	10.10 (4.73)	1.68 (1.54)	14.34	8.42	<0.01	2.32
**OCI-R**	31.58 (16.69)	6.14 (5.26)	8.66	25.44	<0.01	1.99
**CHOCI**	14.00 (12.83)	3.01 (4.23)	5.01	10.99	<0.01	1.13

### Group comparisons on perfectionism measures

The AN group scored significantly higher on both self-report perfectionism measures: the FMPS (PS, CM and DA subscales) and the CPQ. Large effect sizes were detected for both. Table 1 shows mean scores for each group and results of analyses comparing the two groups, along with effect sizes.

#### Text replication task

Compared with the HC group, the AN group took significantly longer to complete the task and produced higher quality copies. Table 2 shows the mean scores for each group on this measure, along with the results of the analysis of group differences and effect sizes. A significant association was found between time taken to complete the task and copy quality in the AN group (r = 0.45, p = <0.01). The correlation between these two measures in the HC group was not significant (r = 0.18, p = 0.18).

**Table 2:** Group differences on performance based perfectionism tasks.

	AN	HC	t	B	p	ES
**Copy task – time (min)** a	22.48 (10.18)	19.19 (7.72)	2.22	3.28	0.03	0.36
**Copy task –mean quality rating (/7)** a	4.85 (0.96)	4.40 (1.02)	2.78	0.45	<0.01	0.45
**N beads left after 60 s** a	1.00 (0, 6)	0.00 (0, 4)	0.73	-	0.46	-
**Time to check (s)** b	11.00 (0, 21)	0.00 (0, 15)	5.26	-	<0.01	0.45
**Control task (s)** b	43.36 (14.72)	43.56 (6.76)	0.95	1.80	0.34	-
**N choosing to check**	48/81 (59%)	33/72 (46%)	2.78	-	0.07	0.30

a Mean (SD);

b Median (IQR).

#### Bead sorting task

Compared with the HC group, there was a trend towards more participants in the AN group choosing to check their work at the end of the task (59% vs 46% in the HC group, p = 0.07). The AN group also spent significantly longer checking than those in the HC group. No difference was found between the two groups on number of beads remaining after 60 seconds. No difference was found on the control task of general performance speed, indicating that observed differences were not simply due to differences in task speed. Table 2 shows the outcomes for each group and results of the analysis.

### Associations between self-report and behavioural measures

Text copy quality was significantly associated with FMPS PS (r = 0.32, p = <0.01), CM (r = 0.26, p = <0.01), DA (r = 0.26, p<0.01), and the CPQ (r = 0.30, p = <0.01). Text replication time was significantly related to FMPS PS (r = 0.17, p = 0.04), CM (r = 0.17, p = 0.04), DA (r = 0.21, p = <0.01) and the CPQ (r = 0.20, p = 0.02). On the bead sorting task, the number of beads remaining at the end of the task was significantly associated with FMPS CM (r = 0.20, p = 0.02) but not with FMPS DA, PS or CPQ score. None of the subscales of the FMPS, or the CPQ were associated with the number of participants in the respective groups choosing to check, nor time spent checking. No associations were found between outcomes on the two behavioural tasks (all t = <1.96). Of all performance based measures, only copy quality was found to be an independent predictor of FMPS CM and PS and CPQ scales. There was a trend towards copy quality also predicting FMPS DA (p = 0.06). No other performance based measure independently predicted outcome on self-reported perfectionism. Table 3 shows the results of the regression analyses.

**Table 3:** Regression models of behavioural predictor variables on self report outcome variables.

	PS			DA			CM			CPQ		
	B	t	p	B	t	p	B	t	p	B	t	p
**Copy time**	0.05	0.80	0.43	0.05	1.34	0.18	0.09	0.95	0.34	0.08	1.03	0.30
**Copy quality**	1.85	3.43	<0.01	0.91	2.68	<0.01	2.24	2.65	<0.01	2.09	3.16	<0.01
**Beads N left**	0.10	1.08	0.28	0.14	2.44	0.02	0.07	0.50	0.62	0.15	1.39	0.17
**Beads check**	−1.18	−0.93	0.36	0.09	0.11	0.92	−0.83	−0.42	0.68	0.27	−0.17	0.87
**Beads checking time**	0.01	0.35	0.73	0.01	0.29	0.78	0.03	0.57	0.57	0.01	0.32	0.75

## Discussion

This study builds upon previous research which has relied upon self-report measures of perfectionism and is the first evidence of elevated perfectionism in AN patients compared with HCs using novel experimental paradigms. Significant differences were also found on self-reported perfectionism between the AN and HC groups, with large effect sizes. This study replicates the findings of previous studies and systematic reviews [6], [8] in a large sample of AN patients including both adolescents and adults.

On a text replication task participants with AN took significantly longer to complete the task and produced significantly higher quality copies. This is in line with a previous study [26] using the same measure in a non-clinical sample where the task was found to distinguish between participants classified as high or low perfectionists. Previous non-clinical research has found that the association between perfectionism and task performance is fully mediated by time taken on the task [25]. However, in contrast, this study found that copy quality, rather than copy time explained unique variance in self-reported perfectionism.

The comparative lack of association between time and task performance in the HC group is of note. Although spending more time on the task was associated with producing a more accurate copy in the AN group, it appears that HC group may have employed a different approach to the task. The AN group produced significantly higher quality copies but this was at the expense of increased time investment. On the bead sorting task, more participants in the AN group chose to check their work (although this just missed significance) and those in the AN group spent significantly longer checking. No difference was found between the two groups in terms of the number of beads remaining at the end of the designated sixty seconds.

Outcomes on the text replication task were significantly associated with two self-report measures of perfectionism: the FMPS and the CPQ. On the bead sorting task, the number of beads remaining was significantly associated with FMPS Concern over Mistakes, suggesting that the speed versus accuracy trade off elicited by this task also taps into aspects of perfectionism captured by existing self-report measures. Although the group difference in the number of beads remaining was not significant, the trend was in the predicted direction. The observed associations between both the FMPS and CPQ and the two performance based measures we used in this study highlight the relevance of these tasks to both research settings - given the debate over measures of perfectionism - and clinical settings – in terms of exploring specific and concrete ideas to address perfectionism in clinical work. The finding that copy quality was the only unique predictor of aspects of self-reported perfectionism considered to be adaptive (FMPS PS) and maladaptive (FMPS CM, DA, CPQ) is interesting. This suggests that whilst better performance is desirable, for participants with AN this performance stems not only from positive achievement striving but is also motivated by attempts to avoid mistakes and by doubts over one's own performance. Whilst those in the AN group produced better copies it was at the expense of increased time invested. This adds to the discussion around perfectionist traits in AN, with regards to whether they are adaptive or maladaptive and further highlights the complex nature of perfectionism. This has important implications not only for researchers but also clinicians, particularly in terms of the need to address the balance between positive achievement striving and maladaptive striving for perfection. It is however, worth considering that other characteristics of the AN group, such as cognitive style, may have contributed to group differences in copy quality. Increased attention to detail is characteristic of AN [40] and may have contributed to the group difference in copy quality, along with differences in perfectionism.

There are a number of limitations to this study. The study included a female only sample, which means that the findings may not be generalizable to males with eating disorders, particularly given research pertaining to gender differences in perfectionism [41]. It would have proved beneficial to include questions concerning approach to the task and motives for approaching the task in this way, in order to try to further investigate subtle differences between adaptive and maladaptive perfectionism which this study has highlighted. Whilst the observed association between behavioural and self-report measures is an important step in the development and validation of more behavioural measures of perfectionism, the limitations of self report measures must be acknowledged in this context. In order to understand and predict personality related behaviour more fully, additional indirect or implicit assessment tools should be adopted alongside (for examples of this type of approach see [42], [43]). It is possible that whilst participants were not told the purpose of the tasks, having completed self report measures on perfectionism, perfectionists may have inferred the nature of the tasks and therefore behaved in line with their egosyntonic personality trait. The development of more implicit measures of personality traits would therefore be beneficial.

The findings of this study are relevant to clinical practice. Not only do they strengthen the evidence for perfectionism in AN but the tasks themselves could be further developed to be utilised within interventions for perfectionism as part of behavioural experiential learning. There is evidence that a cognitive behavioural approach is efficacious in reducing perfectionism in individuals with elevated perfectionism and/or disorders associated with perfectionism, including AN [44], as well as reducing other psychopathology [45]. Simple behavioural tasks such as these, which are specific and concrete but not directly related to participants' own lives and therefore non-threatening, may prove useful within a CBT intervention for perfectionism. They may be used either to illustrate behavioural features of perfectionism or as a target for modification within interventions. This would fit with an approach of focusing initially upon making changes in more manageable areas. Our finding relating to group differences in terms of the association between time and accuracy may also be a pertinent clinical message that more reasonable standards are achievable without an excessive level of striving.

Future studies of interventions targeting perfectionism should consider the use of more experimental and behavioural measures of perfectionism such as those used in this study in addition to self-report measures in order to demonstrate change. Future research should also investigate whether it is possible to modify these behavioural aspects of perfectionism in AN. Existing studies have found experimental evidence that it is possible to manipulate perfectionism in non-clinical samples [26] with this resulting in changes in eating behaviours and attitudes [46], [47]. So called ‘cognitive bias modification’ techniques are attracting considerable interest in a range of psychopathologies [48], [49] and have already shown particular promise in eating disorders [50]. An important next step will be to investigate whether these techniques have utility as clinical interventions in patient populations. The availability of valid and objective behavioural measures with known sensitivity to diagnostic status will be an important addition to the outcomes measures used in this work.
